# Supplementary material for: Conformational plasticity of the HIV-1 gp41 immunodominant region is recognized by multiple non-neutralizing antibodies
Source: Commun Biol. 2022 Mar 31;5:291. doi: 10.1038/s42003-022-03235-w (PMC8971491; doi:10.1038/s42003-022-03235-w)
Supplement: Supplementary file 2 — Supplementary Information [file 42003_2022_3235_MOESM2_ESM.pdf]

Supplementary Information

Conformational plasticity of the HIV-1 gp41 immunodominant region is recognized by multiple non-neutralizing antibodies

Jonathan D. Cook[1,2], Adree Khondker[1], and Jeffrey E. Lee[1,*]

[1]Department of Laboratory Medicine and Pathobiology, Temerty Faculty of Medicine, University of Toronto, Toronto, ON, M5S 1A8, Canada

[2]Present address: Department of Medicine, Temerty Faculty of Medicine, University of Toronto, Toronto, ON, M5S 1A8, Canada

*Correspondence to: J.E.L.

Email: jeff.lee@utoronto.ca

Phone: (416)-978-1060

Fax: (416)-978-5959

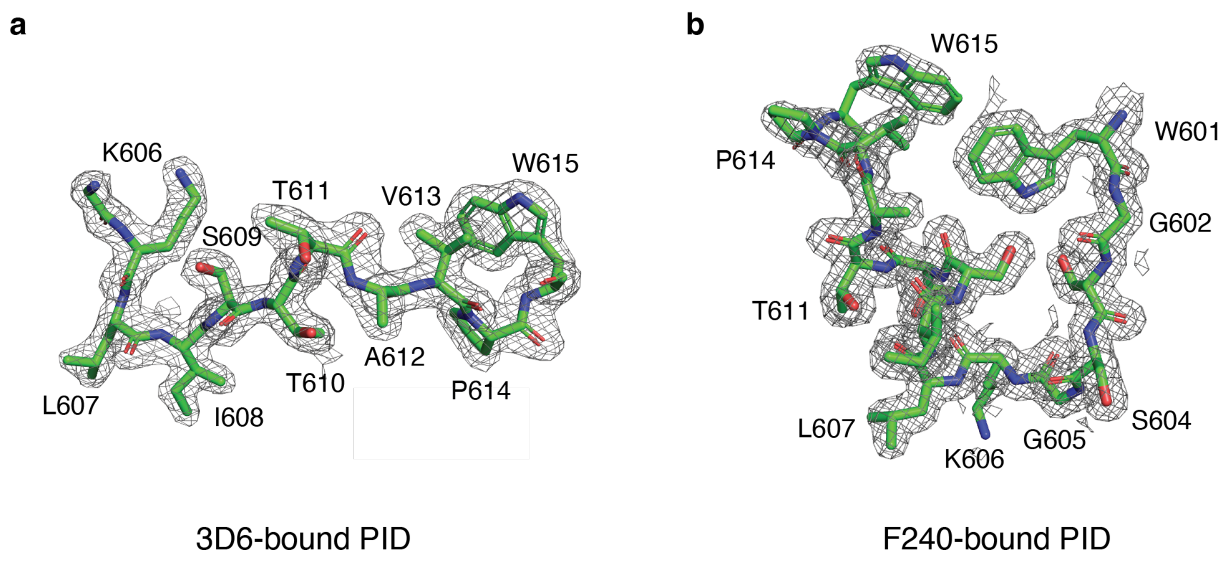

Supplementary Figure 1. Electron density map of Fab 3D6- and F240-bound PID regions. A representative composite omit 2|F_o|-|F_c| electron density map of the **a.** Fab 3D6-bound and **b.** Fab F240-bound PID region contoured at 1σ and superimposed with its final refined model.

```
                                            FR1-IMGT          CDR1-IMGT       FR2-IMGT     CD
                                             (1-26)            (27-38)        (39-55)       (
                                 __________________________ ____________ _________________ __
                                 1       10        20         30         40        50
                                 .........|.........|...... ...|........ .|.........|..... ..
F240 Heavy                       QVQLVQSGG.GVVKPGASLRLACSAS GFTF....TDYY MSWIRQTPGKGLQWLAY IT
M99652 H sapiens IGHV3-11*01 F   QVQLVESGG.GLVKPGGSLRLSCAAS GFTF....SDYY MSWIRQAPGKGLEWVSY IS
                                      Q     V    A    A S           T          T     Q LA   T

                                 R2-IMGT                 FR3-IMGT
                                 56-65)                  (66-104)
                                 ________ _______________________________________
                                  60         70        80        90        100
                                 ..|..... ....|.........|.........|.........|....
F240 Heavy                       KD..GSEK KYADSLQ.GRFAVSRDNANNLVFLQLNTVEDDDTGVYYC AR
M99652 H sapiens IGHV3-11*01 F   SS..GSTI YYADSVK.GRFTISRDNAKNSLYLQMNSLRAEDTAVYYC AR
                                 KD    EK K    LQ    AV     N LVF  L TVEDD  G
```

```
                                            FR1-IMGT          CDR1-IMGT       FR2-IMGT     CD
                                             (1-26)            (27-38)        (39-55)       (
                                 __________________________ ____________ _________________ __
                                 1       10        20         30         40        50
                                 .........|.........|...... ...|........ .|.........|..... ..
F240 kappa                       EFLLTQSPDSLAVTLGETATITCRSS RNILHSLNNKNY LAWYQQRPGQAPKLLVI WA
Z00023 H sapiens IGKV4-1*01 F    DIVMTQSPDSLAVSLGERATINCKSS QSVLYSSNNKNY LAWYQQKPGQPPKLLIY WA
                                 EFLL         T   T  T R    RNI H L            R   A    VI

                                 R2-IMGT                 FR3-IMGT
                                 56-65)                  (66-104)
                                 ________ _______________________________________
                                  60         70        80        90        100
                                 ..|..... ....|.........|.........|.........|....
F240 kappa                       .......S MRVSGVA.DRFSGSG..SGTDFALTISSLQPEDAAVYYC QHYYTTH
Z00023 H sapiens IGKV4-1*01 F    .......S TRESGVP.DRFSGSG..SGTDFTLTISSLQAEDVAVYYC QQYYSTP
                                          M V   A               A       P  A       H  T H
```

```
                                            FR1-IMGT          CDR1-IMGT       FR2-IMGT     CD
                                             (1-26)            (27-38)        (39-55)       (
                                 __________________________ ____________ _________________ __
                                 1       10        20         30         40        50
                                 .........|.........|...... ...|........ .|.........|..... ..
3D6 heavy                        EVQLVESGG.GLVQPGRSLRLSCAAS GFTF....NDYA MHWVRQAPGKGLEWVSG IS
KC713947 H sapiens IGHV3-9*03 F  EVQLVESGG.GLVQPGRSLRLSCAAS GFTF....DDYA MHWVRQAPGKGLEWVSG IS
                                                                    N

                                 R2-IMGT                 FR3-IMGT
                                 56-65)                  (66-104)
                                 ________ _______________________________________
                                  60         70        80        90        100
                                 ..|..... ....|.........|.........|.........|....
3D6 heavy                        WD..SSSI GYADSVK.GRFTISRDNAKNSLYLQMNSLRAEDMALYYC VK
KC713947 H sapiens IGHV3-9*03 F  WN..SGSI GYADSVK.GRFTISRDNAKNSLYLQMNSLRAEDMALYYC AKD
                                  D   S                                           V
```

```
                                            FR1-IMGT          CDR1-IMGT       FR2-IMGT     CD
                                             (1-26)            (27-38)        (39-55)       (
                                 __________________________ ____________ _________________ __
                                 1       10        20         30         40        50
                                 .........|.........|...... ...|........ .|.........|..... ..
3D6 light                        DIQMTQSPSTLSASVGDRVTITCRAS QSI......SRW LAWYQQKPGKVPKLLIY KA
X72813 H sapiens IGKV1-5*03 F    DIQMTQSPSTLSASVGDRVTITCRAS QSI......SSW LAWYQQKPGKAPKLLIY KA
                                                                      R            V

                                 R2-IMGT                 FR3-IMGT
                                 56-65)                  (66-104)
                                 ________ _______________________________________
                                  60         70        80        90        100
                                 ..|..... ....|.........|.........|.........|....
3D6 light                        .......S SLESGVP.SRFSGSG..SGTEFTLTISSLQPDDFATYYC QQYNSYS
X72813 H sapiens IGKV1-5*03 F    .......S SLESGVP.SRFSGSG..SGTEFTLTISSLQPDDFATYYC QQYNSYS
```

Supplementary Figure 2. Comparison of Fab 3D6 and Fab F240 heavy and light chains with the inferred germline. The light and heavy chain variable regions of Fab F240 and Fab 3D6 compared with the inferred germline. Antibody amino acid changes from germline are listed below the sequence alignment and highlighted in bold. The amino acids are labeled according to the Kabat numbering scheme.

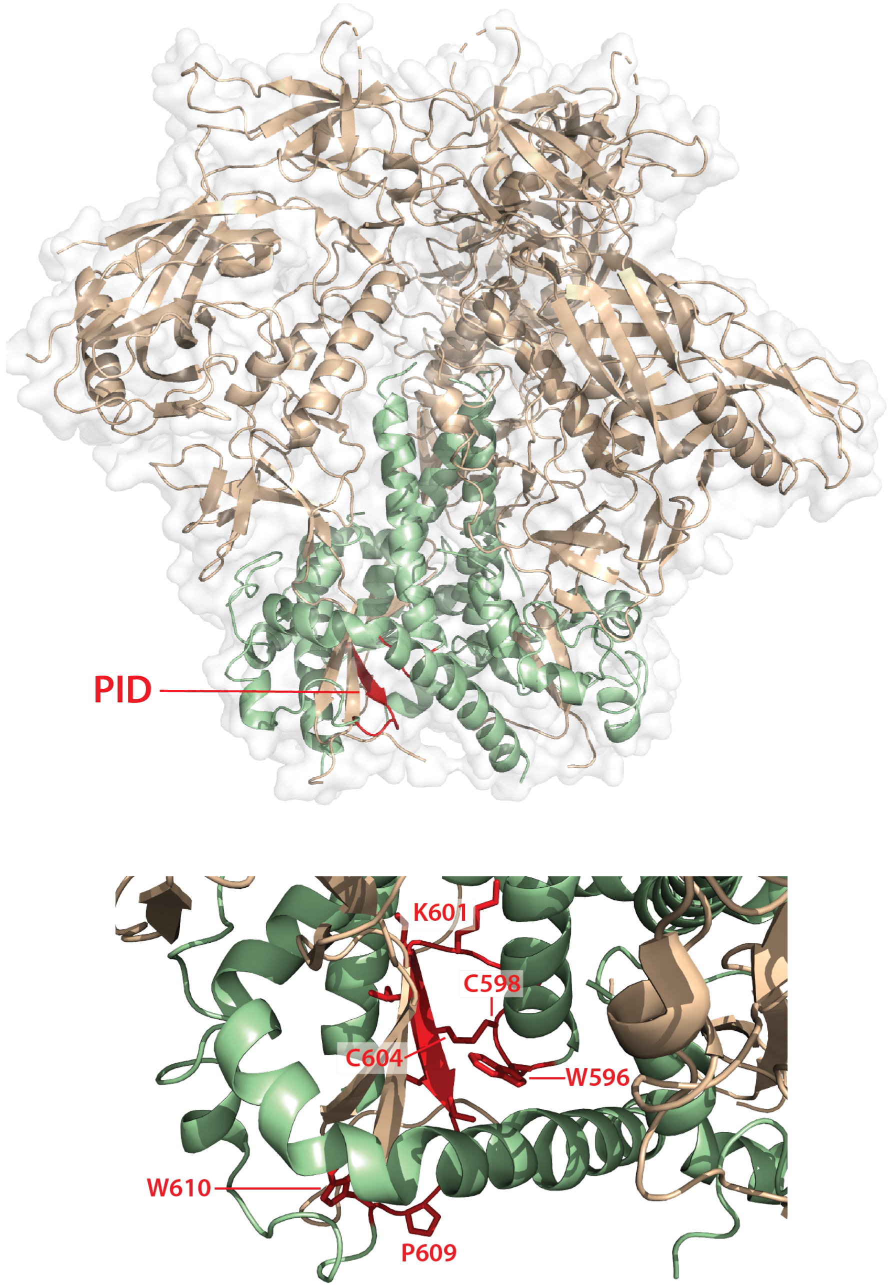

Supplementary Figure 3. Pre-fusion HIV-1 gp140 PID conformation. The crystal structure of pre-fusion HIV-1 SOSIP gp140 bound to human Fab PGT122 and 35O22 (PDB: 4TVP) revealed that PID residues 596-610 (highlighted in red) is buried, with only residue 608-611 partially accessible. Residues 602-606 forms an intermolecular β-sheet with the gp120 subunit. In the Fab F240 and 3D6-bound structures, residues 602-606 forms a short helix and random coil, respectively.

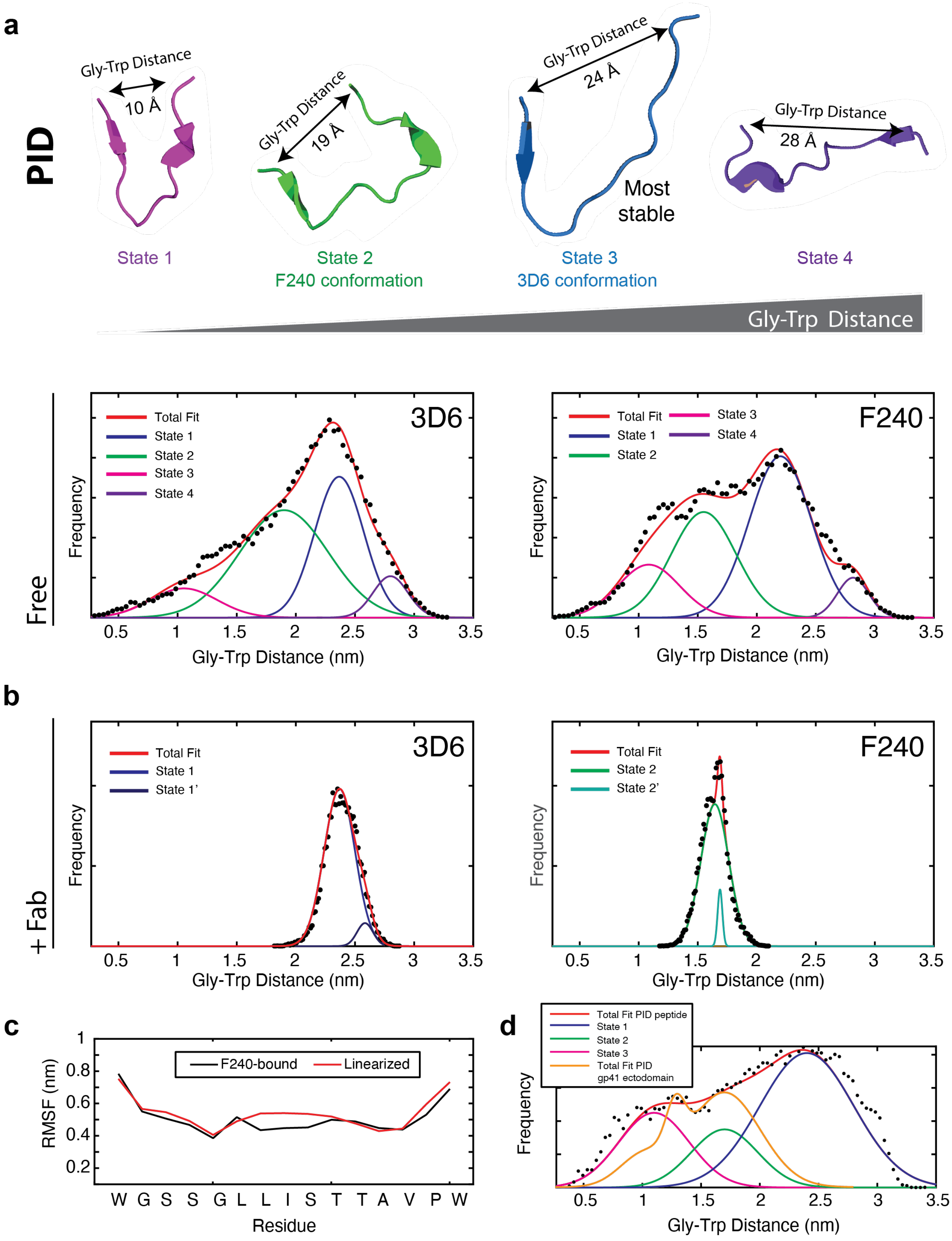
a
PID
Gly-Trp Distance
10 Å
19 Å
24 Å
28 Å
Most stable
State 1
State 2
F240 conformation
State 3
3D6 conformation
State 4
Gly-Trp Distance
Free
3D6
F240
Total Fit
State 1
State 2
State 3
State 4
Frequency
Gly-Trp Distance (nm)
b
+ Fab
State 1'
State 2'
c
F240-bound
Linearized
RMSF (nm)
W G S S G L L I S T T A V P W
Residue
d
Total Fit PID peptide
Total Fit PID
gp41 ectodomain

Supplementary Figure 4. Molecular dynamics simulations of the HIV-1 gp41 PID. a. Analysis of the MD simulations of the PID peptide extracted from the 3D6-bound and F240-bound structures. Raw data showing the frequency of end-to-end distance measurements between Gly600 and Trp610 (black dots) are used to calculate an overall total fit observed in the simulation. Potential metastable states are deconvoluted from the total fit, as estimated by distinct Gaussian distributions. Upon fitting, the analyses revealed four metastable conformations, with end-to-end distances of 1.2, 1.9, 2.4, and 2.8 nm (blue, green, pink and purple curves). The metastable states with 2.4 and 1.9 nm Gly-Trp end-to-end distances agree with the PID conformations observed in the 3D6 and F240-bound structures, respectively. **b.** MD simulations of the PID region bound to Fab 3D6 and Fab F240 showed state selectivity. A single major PID conformation is observed with high frequency when bound to Fab 3D6 or F240. This shows that the PID region is locked into a specific conformation upon binding the Fab. Raw data is shown as black dots with the total fit displayed as the red curve. **c.** Root-mean squared fluctuation (RMSF) analysis of PID region MD simulations starting from either a linearized or F240-bound peptide conformation reveals no difference in positional fluctuation of each residue based on starting conditions. This suggests that the results of MD simulations do not different between using a linearized or Fab-bound conformation of the PID region. **d.** Analysis of PID conformations from MD simulations of the HIV-1 gp41 ectodomain reveal that the Gly-Trp end-to-end distances are observed in the free PID peptide MD simulations as well (Total Fit, State 1, 2 and 3). Raw data showing the frequency of end-to-end distance measurements for the free PID peptide conformation are shown as black dots, with the total fit shown as the red curve. Three metastable conformations (State 1, 2 and 3) are deconvoluted from the total fit. The total fit of the PID region from the MD simulation of the complete gp41 ectodomain (orange curve; also in Figure 6b) reveals a similar conformation to State 2 and 3 in the free PID peptide simulation.

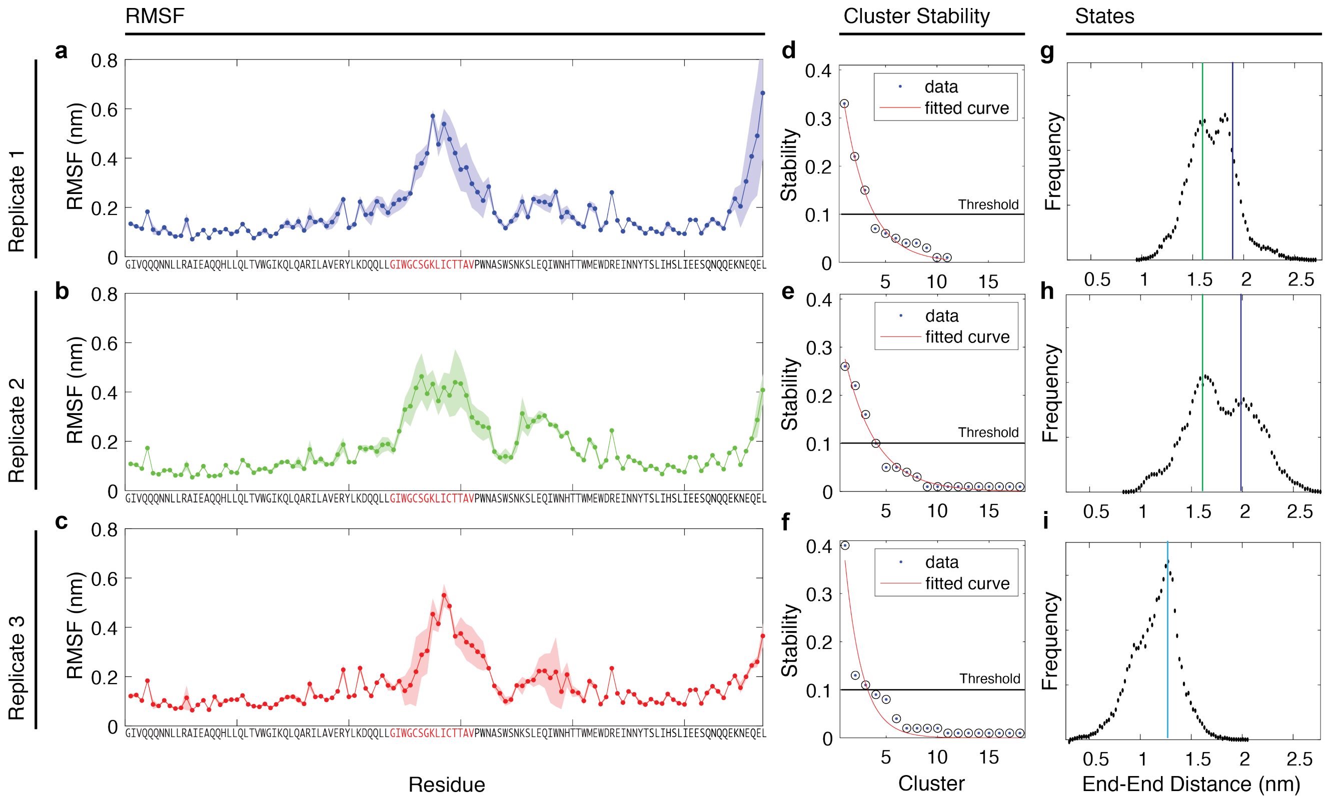

Supplementary Figure 5. Replicates of HIV-1 gp41 ectodomain MD simulation. a-c. RMSF of each residue in three replicate of the complete HIV-1 gp41 ectodomain MD simulation over 100 ns after 100 ns equilibration, **d-f.** Stability (% time spent in conformation) for each cluster from most stable to least stable, fit with decaying exponential, which shows fully the different states found in the PID domain in solution. **g-i.** PID end-to-end distance over the last 100 ns of simulation showing multiple exhibited PID conformations in each replicate. Together, this shows that each replicate has end-to-end distances that correspond with the metastable state conformation end-to-end distances (highlighted lines) found in the full-length HIV-1 gp41 ectodomain suggesting this state reliably occurs in simulation.

F240 heavy chain codon-optimized DNA and protein sequence

```
atggagacagacacactcctgctatgggtactgctgctctgggttccaggttccactggt
 M  E  T  D  T  L  L  L  W  V  L  L  L  W  V  P  G  S  T  G
gaccaggtgcagctggtgcagtcaggtggcggcgtggtgaagccgggcgccagcctgaga
 D  Q  V  Q  L  V  Q  S  G  G  G  V  V  K  P  G  A  S  L  R
cttgcatgtagcgcttccggctttacattcaccgattactatatgtcatggatacgacag
 L  A  C  S  A  S  G  F  T  F  T  D  Y  Y  M  S  W  I  R  Q
acccccggcaaaggcctgcagtggctggcgtacataactaaagatgggtccgagaagaaa
 T  P  G  K  G  L  Q  W  L  A  Y  I  T  K  D  G  S  E  K  K
tacgcggattcactgcaaggaagattcgccgtgtccagagacaacgccaataatctcgtc
 Y  A  D  S  L  Q  G  R  F  A  V  S  R  D  N  A  N  N  L  V
tttctgcagctcaatactgtagaggacgacgataccggcgtgtattactgcgcaagagac
 F  L  Q  L  N  T  V  E  D  D  D  T  G  V  Y  Y  C  A  R  D
gacgggtattacgacagatccggctattacggggtcttcgacctgtggggacagggaatt
 D  G  Y  Y  D  R  S  G  Y  Y  G  V  F  D  L  W  G  Q  G  I
agagtgacagttagcagcgcctcaaccaaagggccctcagtattcccactggcccccagc
 R  V  T  V  S  S  A  S  T  K  G  P  S  V  F  P  L  A  P  S
tctaagtctacaagcggaggcactgccgcgctcggctgtctcgtgaaagattatttccca
 S  K  S  T  S  G  G  T  A  A  L  G  C  L  V  K  D  Y  F  P
gagcctgttactgtgagctggaactccggtgcccttacgtctggcgttcatactttcccg
 E  P  V  T  V  S  W  N  S  G  A  L  T  S  G  V  H  T  F  P
gcggtcctccagtcctctgggctgtactccctctcatcagttgtgaccgtcccaagctca
 A  V  L  Q  S  S  G  L  Y  S  L  S  S  V  V  T  V  P  S  S
agtctgggtactcagacatacatctgtaacgttaatcacaagccatccaataccaaggtg
 S  L  G  T  Q  T  Y  I  C  N  V  N  H  K  P  S  N  T  K  V
gacaaaaaagtggagccaaagtcatgcggccgcctggtgccacggggctcccatcaccat
 D  K  K  V  E  P  K  S  C  G  R  L  V  P  R  G  S  H  H  H
caccatcaccatcatcaccattga
 H  H  H  H  H  H  H  *
```

F240 light chain codon-optimized DNA and protein sequence

```
atggagacagacacactcctgctatgggtactgctgctctgggttccaggttccactggt
 M  E  T  D  T  L  L  L  W  V  L  L  L  W  V  P  G  S  T  G
gacgagtttctgctgacccagagtccagactcactggcagtgactctcggggaaactgcc
 D  E  F  L  L  T  Q  S  P  D  S  L  A  V  T  L  G  E  T  A
acaatcacctgtagaagcagccgcaatattctccacagtctcaacaataagaattacctc
 T  I  T  C  R  S  S  R  N  I  L  H  S  L  N  N  K  N  Y  L
gcatggtaccaacagaggcctggacaggcacctaaactccttgttatttgggcttctatg
 A  W  Y  Q  Q  R  P  G  Q  A  P  K  L  L  V  I  W  A  S  M
cgagtctctggagtggccgacagattctctggttccggctcaggtacagatttcgccctc
 R  V  S  G  V  A  D  R  F  S  G  S  G  S  G  T  D  F  A  L
acaatctccagtctgcagccagaggacgctgcagtgtattattgccagcattactacaca
 T  I  S  S  L  Q  P  E  D  A  A  V  Y  Y  C  Q  H  Y  Y  T
acacacaggacatttggacaaggtacacgggttgagatccgaaggaccgtggctgccccg
 T  H  R  T  F  G  Q  G  T  R  V  E  I  R  R  T  V  A  A  P
tccgtcttcatttttccctccctcagatgaacagttgaaatcaggaacggcttctgtagtt
 S  V  F  I  F  P  P  S  D  E  Q  L  K  S  G  T  A  S  V  V
tgtctcctgaataatttttaccccccgagaagccaaagtgcagtggaaggtggacaacgcc
 C  L  L  N  N  F  Y  P  R  E  A  K  V  Q  W  K  V  D  N  A
ttgcagtcagggaactcacaagaatcagtgactgagcaggacagcaaggacagcacgtat
 L  Q  S  G  N  S  Q  E  S  V  T  E  Q  D  S  K  D  S  T  Y
tcattgagctccacacttacactgagcaaggccgattacgagaagcataaagtgtatgct
 S  L  S  S  T  L  T  L  S  K  A  D  Y  E  K  H  K  V  Y  A
tgtgaggttacccatcagggactgtcctctccagtcacgaagtctttcaaccgggggggag
 C  E  V  T  H  Q  G  L  S  S  P  V  T  K  S  F  N  R  G  E
tgctga
 C  -
```

Supplementary Figure 6. Nucleotide and translated amino acid sequences for Fab F240 heavy and light chains. The native F240 sequence is colored in black, while regions colored in red, green and blue correspond to the Ig-kappa signal peptide, thrombin cleavage tag, and C-terminal 10-histidine purification tag, respectively.

3D6 heavy chain codon-optimized DNA and protein sequence

```
atggagacagacacactcctgctatgggtactgctgctctgggttccaggttccactggt
 M  E  T  D  T  L  L  L  W  V  L  L  L  W  V  P  G  S  T  G
gacgaggtgcagttggtggaatccgggggcgggctggttcaaccgggtcggagtcttagg
 D  E  V  Q  L  V  E  S  G  G  G  L  V  Q  P  G  R  S  L  R
ctttcatgcgcggcaagcgggtttacattcaatgactatgctatgcattgggttcgacag
 L  S  C  A  A  S  G  F  T  F  N  D  Y  A  M  H  W  V  R  Q
gcaccgggaaaaggtcttgaatgggtttccggcatctcatgggattctagttccattgga
 A  P  G  K  G  L  E  W  V  S  G  I  S  W  D  S  S  S  I  G
tatgccgattccgtgaaaggaaggtttacgatctcacgagacaatgccaagaacagcctt
 Y  A  D  S  V  K  G  R  F  T  I  S  R  D  N  A  K  N  S  L
taccttcaaatgaacagtctgcgcgcggaagatatggccctctattattgtgtgaaagga
 Y  L  Q  M  N  S  L  R  A  E  D  M  A  L  Y  Y  C  V  K  G
cgggactactatgactccggcggatacttcaccgttgcctttgatatatggggtcaggga
 R  D  Y  Y  D  S  G  G  Y  F  T  V  A  F  D  I  W  G  Q  G
acaatggtcactgtgtcatcagcatcaacgaagggcccatcagtcttcccgctggccccg
 T  M  V  T  V  S  S  A  S  T  K  G  P  S  V  F  P  L  A  P
agtagtaagtctacttccggcggaactgccgcactgggctgccttgtaaaggactatttt
 S  S  K  S  T  S  G  G  T  A  A  L  G  C  L  V  K  D  Y  F
ccggagccagtcaccgtgagttggaacagtggtgcgcttacatctggagtccatacctt t
 P  E  P  V  T  V  S  W  N  S  G  A  L  T  S  G  V  H  T  F
cctgccgtgttgcagtccagtggcttgtatagcttgtcatctgtggtcacagtaccatct
 P  A  V  L  Q  S  S  G  L  Y  S  L  S  S  V  V  T  V  P  S
tcctcacttgggacacaaacatatatatgtaacgtgaaccataagccatctaataccaaa
 S  S  L  G  T  Q  T  Y  I  C  N  V  N  H  K  P  S  N  T  K
gtggataaaaaagtagagcccaagtcctgcggccgcctggtgccacggggctcccatcac
 V  D  K  K  V  E  P  K  S  C  G  R  L  V  P  R  G  S  H  H
catcaccatcaccatcatcaccattga
H  H  H  H  H  H  H  H  *
```

3D6 light chain codon-optimized DNA and protein sequence

```
atggagacagacacactcctgctatgggtactgctgctctgggttccaggttccactggt
 M  E  T  D  T  L  L  L  W  V  L  L  L  W  V  P  G  S  T  G
gacgacatacaaatgacccaatcccctccactctctcagcctcagtcggagatagggtg
 D  D  I  Q  M  T  Q  S  P  S  T  L  S  A  S  V  G  D  R  V
actattacgtgcagagcgtcacagagcatttccagatggcttgcgtggtaccagcagaag
 T  I  T  C  R  A  S  Q  S  I  S  R  W  L  A  W  Y  Q  Q  K
ccgggcaaagtgccgaaactgctcatctataaggcgagtagcctggagtccggcgtacct
 P  G  K  V  P  K  L  L  I  Y  K  A  S  S  L  E  S  G  V  P
tctaggttctccggctcaggtagtggcacggagttcacacttacaatttcaagtttgcag
 S  R  F  S  G  S  G  S  G  T  E  F  T  L  T  I  S  S  L  Q
ccggacgacttcgccacatactactgtcaacaatataacagttactctttcgggcccgga
 P  D  D  F  A  T  Y  Y  C  Q  Q  Y  N  S  Y  S  F  G  P  G
actaaggtggacattaagcggaccgtagcggcgccttcagtctttatcttcccgccctca
 T  K  V  D  I  K  R  T  V  A  A  P  S  V  F  I  F  P  P  S
gacgaacagttgaagtcaggtactgcgagtgtggtgtgtctccttaataacttttatccg
 D  E  Q  L  K  S  G  T  A  S  V  V  C  L  L  N  N  F  Y  P
cgagaggctaaagtacagtggaaagtcgataacgctctccagagtggcaattcacaggaa
 R  E  A  K  V  Q  W  K  V  D  N  A  L  Q  S  G  N  S  Q  E
tctgtaaccgagcaagattcaaaggatagtacctacagtctgtcatctaccctcactctt
 S  V  T  E  Q  D  S  K  D  S  T  Y  S  L  S  S  T  L  T  L
tctaaagctgactatgaaaaacacaaggtatatgcttgtgaggttacccatcagggactg
 S  K  A  D  Y  E  K  H  K  V  Y  A  C  E  V  T  H  Q  G  L
tcttcacctgtcacgaagtctttcaataggggtgagtgttga
 S  S  P  V  T  K  S  F  N  R  G  E  C  -
```

Supplementary Figure 7. Nucleotide and translated amino acid sequences for Fab 3D6 heavy and light chains. The native 3D6 sequence is colored in black, while regions colored in red, green and blue correspond to the Ig-kappa signal peptide, thrombin cleavage tag, and C-terminal 10-histidine purification tag, respectively.

Supplementary Table 1. Parameters for molecular dynamic simulations

$n_{replicates}$	Peptide/protein	n_{atoms}	Simulation Time (Analyzed Time) /ns	Footnote
2	GYLISTTAVPW	9,651	1000 (700)	-
1	Fab 3D6 + WGSSGYLISTTAVPW	106,989	300 (150)	-
1	WGSSGYLISTTAVPW	8,584	1000 (700)	1
2	WGSSGYLISTTAVPW	49,842	1000 (700)	2
1	Fab F240 + WGSSGYLISTTAVPW	98,052	300 (150)	-
3	HIV-1 gp41	163,534	200 (100)	3

[1]*Initial conformation of the PID peptide was extracted from the complexed Fab structure*
[2]*Initial conformation of the PID peptide was linearized*
[3]*Initial model was based on a homology model created using Phyre2*

Supplementary Movie. Molecular dynamic simulation (100 ns) of the complete HIV-1 gp41 ectodomain in one representative replicate. The video was generated with the PyMol in-build movie generator, with one frame recorded per ns and then overlaid. The PID region is highlighted in red, and an individual chain is highlighted in the chainbow coloring scheme from blue (N-terminus) to red (C-terminus).
