# Supplementary material for: Conformational plasticity of the HIV-1 gp41 immunodominant region is recognized by multiple non-neutralizing antibodies
Source: Commun Biol. 2022 Mar 31;5:291. doi: 10.1038/s42003-022-03235-w (PMC8971491; doi:10.1038/s42003-022-03235-w)
Supplement: Supplementary file 3 — Description of Additional Supplementary Files [file 42003_2022_3235_MOESM3_ESM.pdf]

Description of Additional Supplementary Files

File name: Supplementary Movie
Description: **Molecular dynamic simulation (100 ns) of the complete HIV-1 Page 6 of 15 gp41 ectodomain in one representative replicate.** The video was generated with the PyMol in-build movie generator, with one frame recorded per ns and then overlaid. The PID region is highlighted in red, and an individual chain is highlighted in the chainbow coloring scheme from blue (N-terminus) to red (C-terminus).

File name: Supplementary Data 1
Description: Source data for Figure 1.
