# Supplementary material for: Conformational plasticity of the HIV-1 gp41 immunodominant region is recognized by multiple non-neutralizing antibodies
Source: Commun Biol. 2022 Mar 31;5:291. doi: 10.1038/s42003-022-03235-w (PMC8971491; doi:10.1038/s42003-022-03235-w)
Supplement: Supplementary file 6 — Reporting Summary [file 42003_2022_3235_MOESM6_ESM.pdf]

nature research

Corresponding author(s): Jeffrey E Lee

Last updated by author(s): Feb 20, 2022

Reporting Summary

Nature Research wishes to improve the reproducibility of the work that we publish. This form provides structure for consistency and transparency in reporting. For further information on Nature Research policies, see our Editorial Policies and the Editorial Policy Checklist.

Statistics

For all statistical analyses, confirm that the following items are present in the figure legend, table legend, main text, or Methods section.

n/a	Confirmed	
☐	☒	The exact sample size (n) for each experimental group/condition, given as a discrete number and unit of measurement
☐	☒	A statement on whether measurements were taken from distinct samples or whether the same sample was measured repeatedly
☒	☐	The statistical test(s) used AND whether they are one- or two-sided<br>*Only common tests should be described solely by name; describe more complex techniques in the Methods section.*
☒	☐	A description of all covariates tested
☒	☐	A description of any assumptions or corrections, such as tests of normality and adjustment for multiple comparisons
☒	☐	A full description of the statistical parameters including central tendency (e.g. means) or other basic estimates (e.g. regression coefficient) AND variation (e.g. standard deviation) or associated estimates of uncertainty (e.g. confidence intervals)
☒	☐	For null hypothesis testing, the test statistic (e.g. F, t, r) with confidence intervals, effect sizes, degrees of freedom and P value noted<br>*Give P values as exact values whenever suitable.*
☒	☐	For Bayesian analysis, information on the choice of priors and Markov chain Monte Carlo settings
☒	☐	For hierarchical and complex designs, identification of the appropriate level for tests and full reporting of outcomes
☒	☐	Estimates of effect sizes (e.g. Cohen's d, Pearson's r), indicating how they were calculated

Our web collection on statistics for biologists contains articles on many of the points above.

Software and code

Policy information about availability of computer code

Data collection	MxLIVE (Canadian Light Source; 08ID-1 beamline data collection software)
Data analysis	All programs are either commercially or freely available for academics. No custom algorithms or software were used.<br>X-ray data reduction and scaling- XDS and Aimless (CCP4 v7.1)<br>Molecular Replacement- PHENIX.Phaser (v1.19.2-4158)<br>Model building- PHENIX.autobuild (v1.19.2-4158)<br>Model building- Coot (v0.9.5)<br>Model refinement- PHENIX.refine (v1.19.2-4158)<br>MD simulation- GROMACS (v5.1.4)<br>MD simulation analysis- MATLAB with SpectralTools and Mfit4 plugins (v2019b)<br>Molecular visualization and representation- PyMOL (v2.5.2) and VMD (v1.9.3)

For manuscripts utilizing custom algorithms or software that are central to the research but not yet described in published literature, software must be made available to editors and reviewers. We strongly encourage code deposition in a community repository (e.g. GitHub). See the Nature Research guidelines for submitting code & software for further information.

Data

Policy information about availability of data

All manuscripts must include a data availability statement. This statement should provide the following information, where applicable:

- Accession codes, unique identifiers, or web links for publicly available datasets
- A list of figures that have associated raw data
- A description of any restrictions on data availability

Atomic coordinates and structure factors for patient-derived Fabs with and without the HIV-1 gp41 PID peptide have been deposited in the Protein Data Bank with the accession codes as follows: apo Fab 3D6: 7N07; Fab 3D6-PID complex: 7N08; apo Fab F240: 7N04; Fab F240-PID complex: 7N05. Further information and reasonable requests for resources and reagents should be directed to the Corresponding Author, Jeffrey E. Lee (jeff.lee@utoronto.ca).

Field-specific reporting

Please select the one below that is the best fit for your research. If you are not sure, read the appropriate sections before making your selection.

☒ Life sciences ☐ Behavioural & social sciences ☐ Ecological, evolutionary & environmental sciences

For a reference copy of the document with all sections, see nature.com/documents/nr-reporting-summary-flat.pdf

Life sciences study design

All studies must disclose on these points even when the disclosure is negative.

Sample size	This study is focused on crystal structures and MD simulations, sample size is not applicable.
Data exclusions	No data was excluded from the study
Replication	ELISA and MD simulations were performed in triplicates, and confirmed to be reproducible.
Randomization	No animals/organisms were used in our study, thus no randomization was performed.
Blinding	The study is focused on crystal structures and MD simulations, no blinding is necessary.

Reporting for specific materials, systems and methods

We require information from authors about some types of materials, experimental systems and methods used in many studies. Here, indicate whether each material, system or method listed is relevant to your study. If you are not sure if a list item applies to your research, read the appropriate section before selecting a response.

Materials & experimental systems

n/a	Involved in the study	
☐	☒	Antibodies
☐	☒	Eukaryotic cell lines
☒	☐	Palaeontology and archaeology
☒	☐	Animals and other organisms
☒	☐	Human research participants
☒	☐	Clinical data
☒	☐	Dual use research of concern

Methods

n/a	Involved in the study	
☒	☐	ChIP-seq
☒	☐	Flow cytometry
☒	☐	MRI-based neuroimaging

Antibodies

Antibodies used	Fab F240 and Fab 3D6
Validation	These Fab were expressed, purified and characterized in this study. Fab F240 and 3D6 were validated to bind the HIV-1 PID region.

Eukaryotic cell lines

Policy information about cell lines

Cell line source(s)	ATCC (CRL-3216)

Authentication	None of the cell lines were authenticated in house. Cell lines were only used for the expression of Fab 3D6 and F240
Mycoplasma contamination	Cell lines were not tested for mycoplasma contamination, as they were only used for the expression of Fab 3D6 and F240. Fabs that were expressed were fully characterized for their identity.
Commonly misidentified lines (See ICLAC register)	none
